# Development of an *In Vitro* Assay and Demonstration of *Plasmodium berghei* Liver-Stage Inhibition by TRAP-Specific CD8^+^ T Cells

**DOI:** 10.1371/journal.pone.0119880

**Published:** 2015-03-30

**Authors:** Rhea J. Longley, Karolis Bauza, Katie J. Ewer, Adrian V. S. Hill, Alexandra J. Spencer

**Affiliations:** The Jenner Institute, University of Oxford, Oxford, United Kingdom; INSERM, FRANCE

## Abstract

The development of an efficacious vaccine against the *Plasmodium* parasite remains a top priority. Previous research has demonstrated the ability of a prime-boost virally vectored sub-unit vaccination regimen, delivering the liver-stage expressed malaria antigen TRAP, to produce high levels of antigen-specific T cells. The liver-stage of malaria is the main target of T cell-mediated immunity, yet a major challenge in assessing new T cell inducing vaccines has been the lack of a suitable pre-clinical assay. We have developed a flow-cytometry based *in vitro* T cell killing assay using a mouse hepatoma cell line, Hepa1-6, and *Plasmodium berghei* GFP expressing sporozoites. Using this assay, *P*. *berghei* TRAP-specific CD8^+^ T cell enriched splenocytes were shown to inhibit liver-stage parasites in an effector-to-target ratio dependent manner. Further development of this assay using human hepatocytes and *P*. *falciparum* would provide a new method to pre-clinically screen vaccine candidates and to elucidate mechanisms of protection *in vitro*.

## Introduction

Malaria, caused by the parasite *Plasmodium*, remains a serious public health concern particularly in developing countries where it is a significant cause of morbidity and mortality [1], and promotes the cycle of poverty. Vaccines are considered one of the most cost-effective public health tools, yet a highly efficacious vaccine against malaria has yet to be achieved. One promising avenue has been the use of viral vectored vaccines to deliver the pre-erythrocytic antigen thrombospondin-related adhesion protein (TRAP) [2], also known as SSP2. This vaccination regimen provided 21% sterile efficacy and a higher rate of partial efficacy manifest as delay to patency in a controlled human malaria infection trial; protection was associated with interferon-gamma (IFN-γ) producing CD8^+^ T cells [3], suggesting TRAP might be the target of cell-mediated immunity at the liver-stage of infection. TRAP is a type 1 transmembrane protein essential for attachment and invasion of both the mosquito salivary gland and the liver, whilst also contributing to the sporozoite gliding motility [4–8]. TRAP has also been demonstrated to provide protection in a number of murine models, utilizing both the *P*. *falciparum* TRAP construct used in clinical trials [9, 10] and independently with the *P*. *vivax* ortholog [11].

Unfortunately, there is currently no standardized assay available to confirm the mechanism by which IFN-γ producing CD8^+^ T cells are associated with protection. *P*. *falciparum* cannot naturally infect small animals, and even if transgenic parasites or humanized mice are utilized to overcome this barrier, the murine immune system is still not equivocal to that of humans, particularly in terms of major histocompatibility complex (MHC) restriction and immunodominance. Hence, the assay of choice to assess the functionality of human T cells against *P*. *falciparum* liver-stages is an *in vitro* model. Whilst such an assay does not currently exist, similar methodologies have been used extensively to assess the effect of antibodies on liver-stage growth and development [12–18], and this assay has recently been standardized [19]. However, assays measuring cellular inhibition are more complicated given the additional need for MHC antigen matching between the hepatocytes and the effector T cells.

Whilst murine *in vitro* cellular assays have been reported in the past [20–24], they have not been used regularly to measure cellular inhibition. Hoffman *et al*. used splenocytes from mice vaccinated with irradiated sporozoites [20], whilst Weiss *et al*. used splenocytes from mice vaccinated with irradiated sporozoites that had been pre-cultured with the *P*. *yoelii* (Py) circumsporozoite protein (CSP) peptide and interleukin 2 (IL-2), to determine whether T cells directed against the PyCSP peptide could specifically inhibit liver-stage parasites [23]. Renia *et al*. utilized both cells from lymph nodes taken from mice vaccinated with a PyCSP synthetic peptide [22] and a CD4^+^ T cell clone generated from the same vaccination regimen [21]. Trimnell *et al*. more recently assessed the inhibitory activity of CD8^+^ enriched cells from both the spleens and livers of mice immunized with *P*. *yoelii* genetically attenuated parasites (PyUIS4−/−) [24]. All studies were able to detect inhibition of liver-stage parasites by fixation, staining and manual counting, and have contributed substantially to our understanding of the role of T cells during liver-stage malaria infection. Given our recent advances in the development of a liver-stage vaccine, it is timely to revisit and reassess the feasibility and utility of such an assay.

In this study we have aimed to further develop such an *in vitro* assay, focusing first on a murine model utilizing *P*. *berghei* (Pb), in order to simplify MHC matching between effector and target cells. The *P*. *berghei* ortholog of PfTRAP (PbTRAP) is also protective against homologous challenge in a C57BL/6 mouse model, with CD8^+^ T cells implicated in protection [25]. We have used our assay to demonstrate *P*. *berghei* TRAP-specific CD8^+^ T cell inhibition of liver-stage parasites in an effector-to-target (E:T) ratio dependent manner. As we have demonstrated the feasibility of this assay using a simplified murine model, we now plan to further develop this assay using human hepatocytes and *P*. *falciparum*. If successful, this will provide a new method to pre-clinically screen *P*. *falciparum* vaccine candidates and to elucidate mechanisms of protection *in vitro*, without reliance on murine models.

## Materials and Methods

### Experimental animals and parasites

Female C57BL/6 mice (H-2^b^) of at least six weeks of age (Harlan, UK) were used in all experiments. *P*. *berghei* blood-stage parasites expressing the green fluorescent protein (GFP) under control of the elongation factor 1α promoter were provided by Prof. Robert Sinden at Imperial College, London [26]. Sporozoites were obtained by dissection and homogenization of salivary glands from infected *Anopheles stephensi* mosquitoes.

### Ethics statement

All animal work was conducted in accordance with the UK Animals (Scientific Procedures) Act 1986 and approved by the University of Oxford Animal Care and Ethical Review Committee for use under Project License PPL 30/2414 or 30/2889. Animals were group housed in individually ventilated cages under specific pathogen free conditions, with constant temperature, humidity and with a 12:12 light-dark cycle (8am to 8pm). For induction of short-term anesthesia, animals were either injected intramuscularly (i.m.) with rompun and Domitor or anaesthetized using vaporized IsoFlo. All animals were humanely sacrificed at the end of each experiment by an approved Schedule 1 method (cervical dislocation). All efforts were made to minimize suffering.

### Labeling and infection of the Hepa1-6 cell line

The murine Hepa1-6 cell line (C57L hepatoma, H-2^b^) (European Collection of Cell Cultures) [27–29] was propagated in supplemented DMEM (2mM L-glutamine, 100U penicillin, 100μg streptomycin, 50μm 2-mercaptoethanol and 10% fetal calf serum (FCS)). In order to discriminate between hepatocytes and added splenocytes, Hepa1-6 cells were first labeled with the membrane dye Vybrant DiD (Life Technologies) according to the manufacturer’s instructions. 5x10^4^ labeled cells were added per well of a 96-well flat bottom plate and left to form a monolayer overnight, prior to infection with 40 000 *P*. *berghei* GFP sporozoites (resulting in the most reliable and consistent levels of infection from the recommended range [30]). Plates were centrifuged at 500xg for five minutes and incubated for three to six hours prior to addition of cells, to allow time for the sporozoites to invade [31]. Experimental wells were performed at least in duplicate, and in triplicate where possible. If no cells were added, the medium was changed at three hours post-infection to reduce potential contamination.

### Assessment of infectivity via flow cytometry

After 24 hours at 37°C, cells were incubated with trypsin for four minutes prior to collection into 10% FCS in phosphate buffered saline, centrifugation and resuspension in 2% FCS in phosphate buffered saline, as previously described [32]. Immediately prior to acquisition on a LSR II flow cytometer (BD Biosciences), 4',6-diamidino-2-phenylindole dihydrochloride (DAPI, final concentration 1μg/ml, Sigma Aldrich) was added to stain dead cells. Cells were gated on size and doublet negativity, followed by exclusion of dead cells. The hepatocytes were selected by DiD positivity and GFP positive hepatocytes were considered infected. Data were analysed using FlowJo (Tree Star Inc.). The background GFP fluorescence was always subtracted based on measurements from at least six uninfected wells.

### *P*. *berghei* TRAP vaccine

*P*. *berghei* TRAP (PbTRAP) (NCBI AAB63302.1) was expressed in the viral vectors chimpanzee adenovirus 63 (ChAd63) and modified vaccinia virus Ankara (MVA) following previously described methods [33, 34]. The vaccines were administered in a prime-boost regimen with 1x10^8^ infectious units ChAd63 administered i.m. followed at least eight weeks later by 1x10^6^ pfu MVA (always one week prior to splenocyte harvest).

### Enrichment of CD8^+^ splenocytes

One-week post-MVA boost, spleens were harvested and single cell suspensions were prepared by passing splenocytes through a 70μm cell strainer followed by incubation with ammonium-chloride-potassium lysate buffer to remove red blood cells. Splenocytes were enriched for CD8^+^ cells by negative depletion using MACS Anti-Biotin MicroBeads and a biotin antibody cocktail, according to the manufacturer’s instructions. Briefly, splenocytes were first labeled with a biotin-antibody cocktail containing monoclonal antibodies against CD4 (clone GK1.5), CD11b (clone M1/70), CD11c (clone N418), CD19 (clone M1319-1), CD45R (B220) (clone RA3-6B2), CD49b (clone DX5) and MHC Class II (clone M5/114.15.2) (all from BioLegend), followed by addition of Anti-Biotin MicroBeads before separation using a MACS Separator and LD Column. Purity of the fractions was determined by staining with anti-CD8α-FITC (clone 53–6.7, eBiosciences) and compared with unfractionated splenocytes.

### Intracellular cytokine staining (ICS)

To determine the frequency of TRAP specific cells, an aliquot of pre-enrichment splenocytes were stimulated for six hours with 5μg/ml of a single pool of PbTRAP peptides (60 20mers overlapping by 10aa), 1μg/ml GolgiPlug (BD Biosciences), and CD107a-PE (clone 1D4B). The PbTRAP pool includes the previously mapped CD8^+^ epitope PbTRAP_130_ [25]. CD107a is a glycoprotein present in the membrane of cytotoxic granules and can be detected on the cell surface following degranulation; it is therefore an indirect marker of cytotoxic activity and identifies cells with this capability. Degranulation is an obligatory process for subsequent cytotoxic killing mediated by release of perforin or granzymes, and CD107a expression has been shown to represent cells capable of cytotoxicity in an antigen-specific manner [35].

Cells were subsequently surface stained with CD16/32 (clone 93), CD4-eFluor 450 (clone RM4-5) and CD8α-PerCPCy5.5 (clone 53–6.7) followed by fixation with 10% neutral buffered formalin (containing 4% paraformaldehyde) (Sigma Aldrich). Staining of intracellular cytokines was achieved using tumour necrosis factor-alpha (TNF-α)-FITC (clone MP6-XT22), IL-2-PeCy7 (clone JES6-5H4) and IFN-γ-APC (clone XMG1.2) diluted in Perm/Wash buffer (BD Biosciences). All antibodies were purchased from either eBiosciences or BD Biosciences. Data were acquired on a LSRII flow cytometer and analysed using FlowJo. Cells were gated on size (FSC vs SSC) and exclusion of doublet cells (FSC-A vs FSC-H), followed by gating on CD4 or CD8 positive cells and identification of cytokine or CD107a positivity. Background responses were measured from unstimulated samples (without peptide) and these were subtracted from the overall response in stimulated samples.

### *In vitro* T cell killing assay

DiD labeled Hepa1-6 cells were infected with *P*. *berghei* GFP sporozoites. Three to six hours later CD8^+^ enriched splenocytes from PbTRAP vaccinated mice were added to the assay at various ratios, typically 2x10^6^ total splenocytes. Control wells contained CD8^+^ enriched splenocytes from naïve or vector control vaccinated mice (ChAd63-MVA luciferase), as stated. Cells were incubated for 24 hours, prior to analysis by flow cytometry. The E:T ratio was back calculated using the purity of the CD8^+^ enrichment and the percentage of antigen-specific cells measured via ICS. The percentage inhibition was calculated using the following formula [20]:

% Inhibition = 1-(test well/average of control wells) x100.

### Statistical analysis

Prism version 5 (Graphpad, USA) was used for all analyses. Non-parametric data are shown as the median ± the interquartile range, or with individual data points plotted. The Mann Whitney test was used to compare the medians of two groups of non-parametric data, and correlations tested using Spearman’s rank correlation. The significance threshold was 0.05.

## Results

### Infectivity and measurement of liver-stage *P*. *berghei* cultures

**Fig 1:**
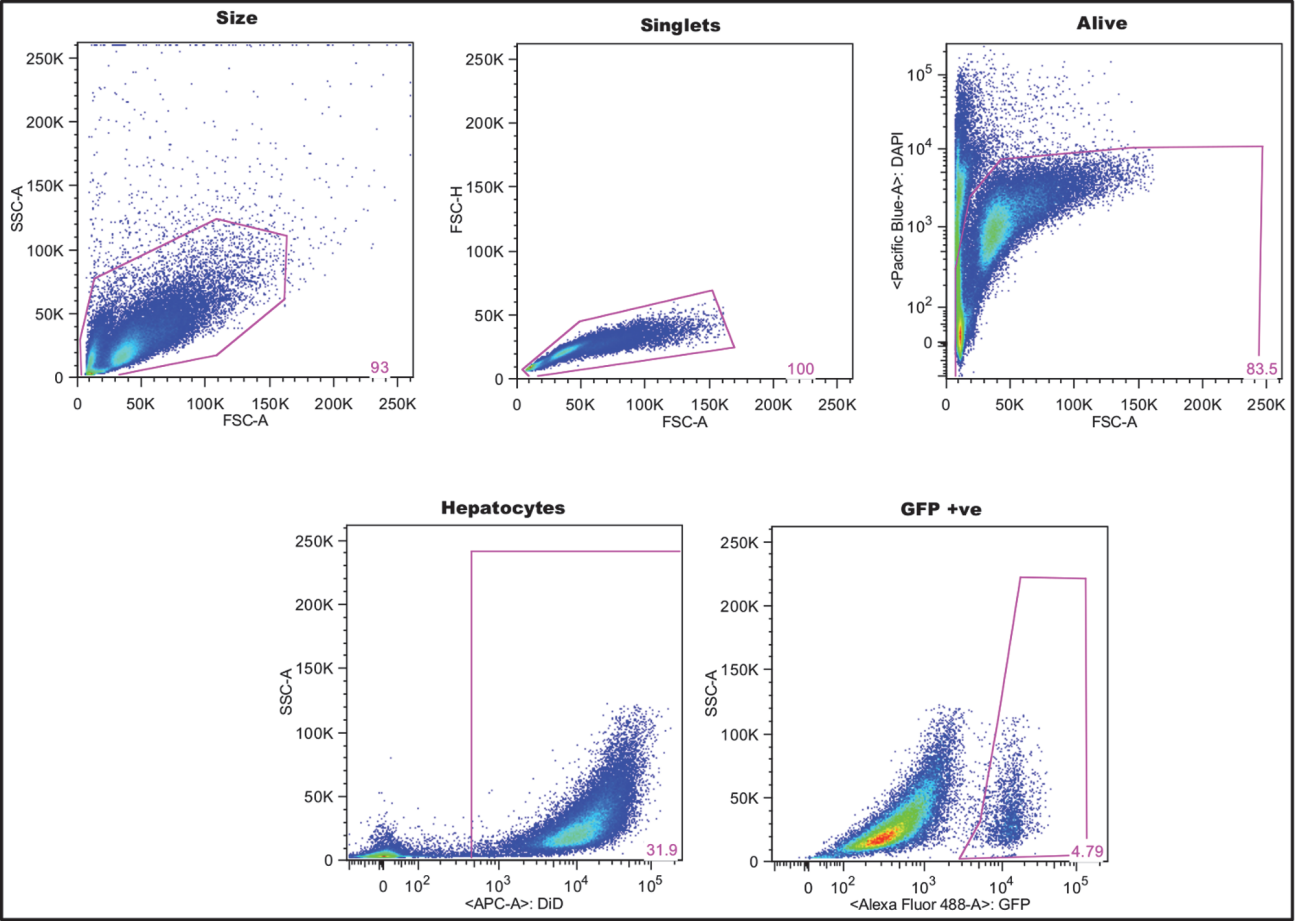
The gating strategy used to define infected hepatoma cells. Cells were gated by size, followed by exclusion of doublets. Viable cells were selected based on the absence of DAPI staining. Hepatoma cells were labeled with the Vybrant DiD membrane dye prior to seeding, thus enabling identification with DiD fluorescence. Finally, the hepatoma cells that were GFP positive were selected. Uninfected hepatoma cells were run as a control to guide gating of the population of GFP^+^ cells.

As various methodologies, cell lines and parasites had been previously used [20–23] we first needed to establish a reliable and standard method for infecting and measuring infection in our laboratory. Isolation and culture of primary murine hepatocytes requires technical expertise and highly specialized reagents and protocols, therefore we chose to use a cell line, Hepa1-6, instead. Using a cell line reduces the variability in the quality of cells, but limits the assay to H-2 backgrounds on which such cell lines are available. We chose to use the Hepa1-6 cell line given the relatively high infectivity of *P*. *berghei* sporozoites previously achieved [36], and given our target vaccine PbTRAP induces a good response in C57BL/6 mice of the same H-2 background (see ‘Characterization of splenocytes’ below). We defined our gating strategy based on selection of viable hepatocytes, with positive GFP expression representing *P*. *berghei* infected cells (Fig. 1); this method of measuring infectivity has previously been shown to correlate with the number of infected cells as per traditional microscopy or PCR [32].

**Fig 2:**
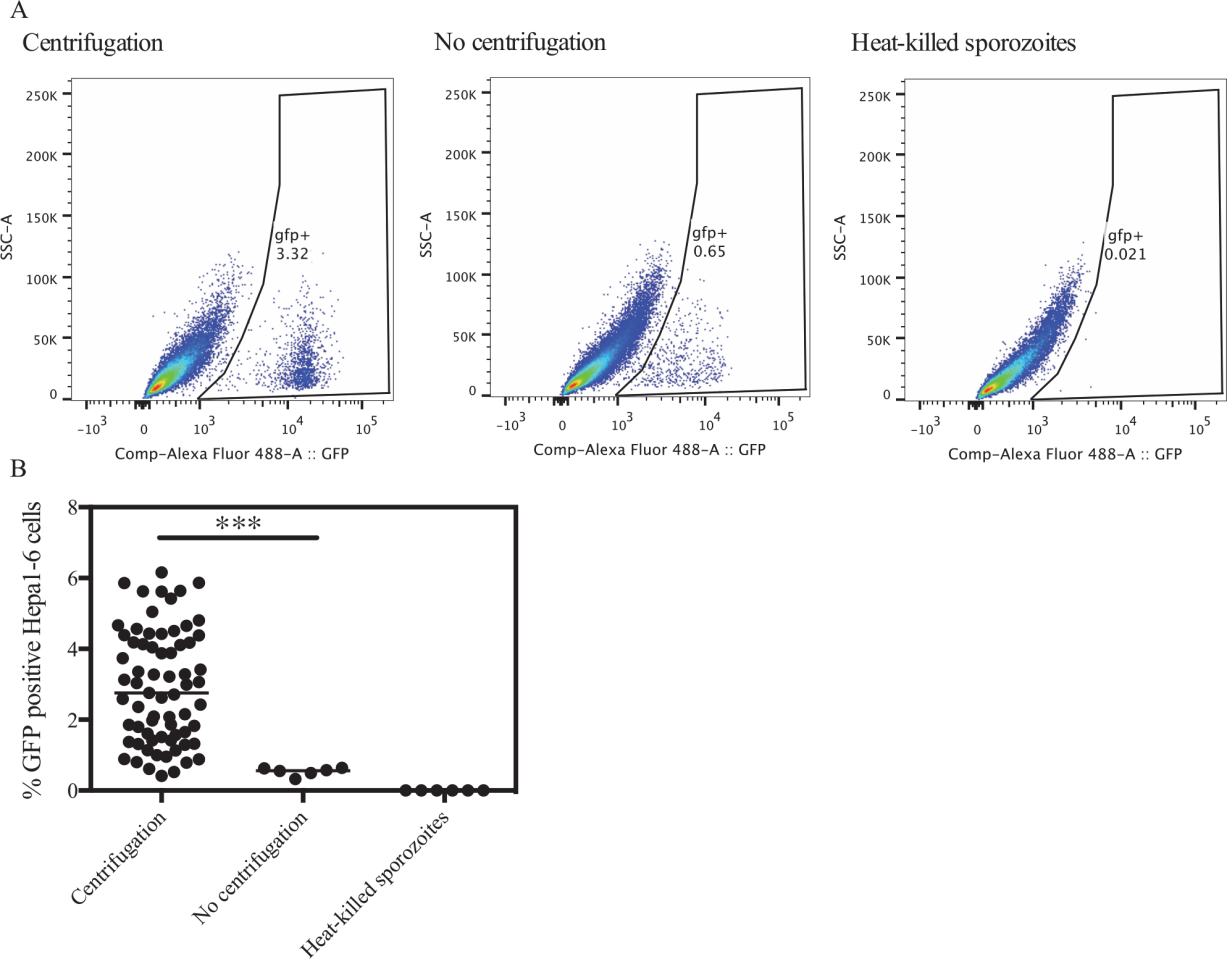
The infectivity of the Hepa1-6 cell line. 40 000 *P*. *berghei* GFP sporozoites were added per well with (n = 69) or without (n = 6) centrifugation. To determine whether dead or aborted sporozoites maintained expression of GFP, sporozoites were heat-killed for 20 minutes at 95°C prior to infection of Hepa1-6 cells (n = 6). 24 hours post-infection cells were harvested and run on a flow cytometer. (A) Representative example of the flow cytometry plots. (B) Data from multiple experiments was pooled and results are expressed as the percentage of GFP positive viable Hepa1-6 cells. The effect of centrifugation on infectivity was assessed using the Mann Whitney test, *** p = 0.0001.

Centrifugation of the plates immediately after addition of sporozoites was shown to increase infectivity from a median of 0.56% to 2.76% (Mann Whitney test, p = 0.0001) (Fig. 2), consistent with recent reports [19, 30] and with other infectious agents [37, 38]. We also confirmed that dead sporozoites no longer express GFP by the addition of heat-killed sporozoites into the assay (Fig. 2).

### Characterization of splenocytes and their addition to the *in vitro* assay

**Fig 3:**
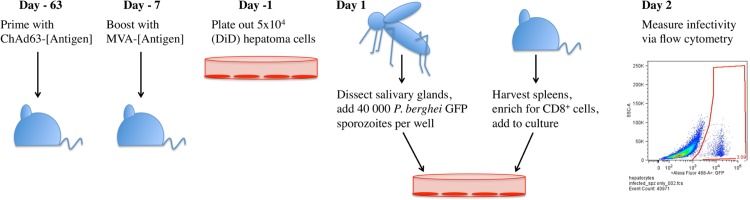
A schematic representation of the murine *in vitro* T cell killing assay. C57BL/6 mice were vaccinated with a ChAd63-MVA prime-boost regimen using vaccines expressing *P*. *berghei* TRAP. Six days post-MVA boost, Hepa1-6 cells were labeled with the membrane dye Vybrant DiD and seeded into a 96-well flat bottom plate. The following day salivary glands were dissected from *P*. *berghei* GFP infected mosquitoes, sporozoites isolated and 40 000 added per well to the Hepa1-6 cell cultures, followed by centrifugation at 500xg for five minutes. Subsequently, spleens from vaccinated mice were enriched for CD8^+^ cells via negative depletion prior to addition to the sporozoite infected Hepa1-6 cell cultures approximately three hours post-infection. Splenocytes were also harvested from vector control vaccinated mice and treated in the same way, to determine the effect of non-specific killing. Plates were then incubated for approximately 24 hours prior to trypsin treatment to harvest cells for acquisition on the flow cytometer.

**Fig 4:**
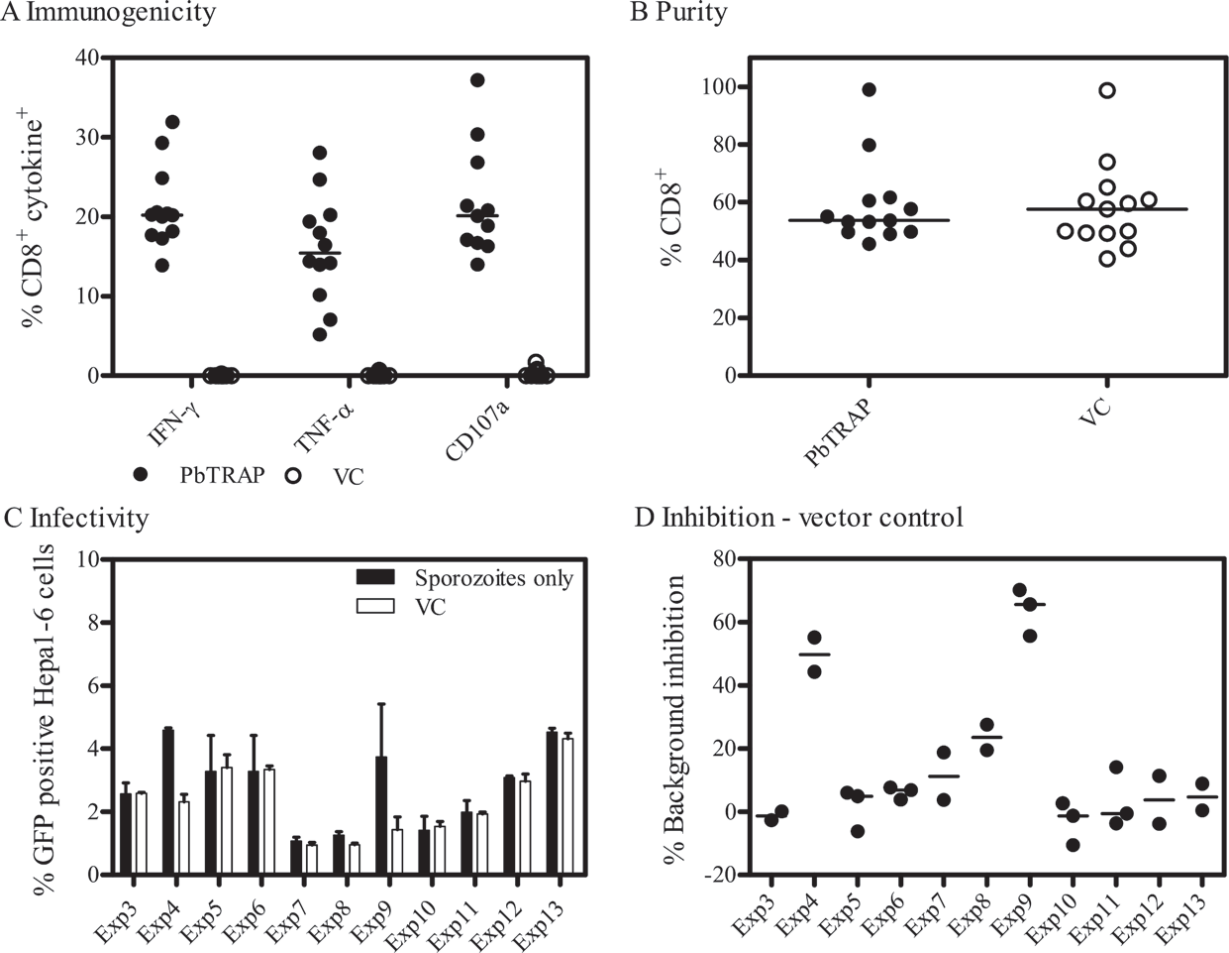
Characterization of splenocytes used in the *in vitro* assays. (A) Cellular immunogenicity of ChAd63-MVA *P*. *berghei* TRAP in C57BL/6 mice. Each data point represents splenocytes from two mice pooled together, with twelve pairs in total that were used in thirteen assays (one pair provided enough cells for two experiments). Cellular immunogenicity was assessed by ICS, after six hours stimulation with a pool of *P*. *berghei* TRAP peptides. Vector control mice were vaccinated with ChAd63-MVA luciferase and treated identically to the experimental mice. Results are expressed as the percentage of CD8^+^ cells, with median and individual data points shown. (B) Prior to addition of the splenocytes into the *in vitro* assays, samples were enriched for CD8^+^ cells. Results are expressed as the percentage of CD8^+^ cells out of total splenocytes, with both median and individual data points shown. (C) In each *in vitro* assay conducted, CD8^+^ enriched splenocytes from vector control vaccinated mice were included in the assay, along with wells containing sporozoites only, to act as controls. Results are expressed as the percentage infectivity, with the median shown for each experiment and error bars representing the interquartile range. (D) These controls allowed calculation of the background level of non-specific inhibition. Results are expressed as the percentage inhibition of splenocytes from vector control vaccinated mice compared to sporozoite only wells (no splenocytes), with median and individual data points shown for each experiment. In Exp1 and Exp2 sporozoite only wells were not included and hence the background inhibition could not be calculated.

After establishment of the liver-stage *P*. *berghei* cultures, we developed a cellular inhibition assay (Fig. 3) based on those previously described. Given TRAP is an antigen of keen interest for a pre-erythrocytic malaria vaccine [3] we wanted to determine the ability of TRAP-specific cells to inhibit liver-stage parasites *in vitro*. Thirteen experiments were conducted, using splenocytes from twelve sets of independently vaccinated mice (Table 1). After vaccination of these mice in a prime-boost regimen, the median PbTRAP-specific CD8^+^ IFN-γ^+^ response in the spleen was 20.22% of total CD8^+^ cells, CD8^+^ TNF-α^+^ 15.43% and CD8^+^ CD107a^+^ 20.10%, compared to no PbTRAP-specific response from naïve or vector control vaccinated mice (ChAd63-MVA luciferase) (Fig. 4A).

**Table 1:** Outline and characteristics of splenocytes used in the thirteen *in vitro* assays performed.

Exp.	Control	Total splenocytes	Splenocytes: hepatocytes	E:T ratio^a^
1	Luciferase	5.0E+05	10:1	58:1^b^
2	Luciferase	1.7E+06	34:1	209:1^b^
3	Naïve	2.0E+06	40:1	255:1
4	Luciferase	2.0E+06	40:1	169:1
5	Luciferase	2.0E+06	40:1	148:1
6	Luciferase	1.0E+06	20:1	74:1
7	Luciferase	2.0E+06	40:1	400:1
8	Luciferase	2.0E+06	40:1	180:1
9	Luciferase	2.0E+06	40:1	95:1
10	Luciferase	2.0E+06	40:1	243:1
11	Luciferase	2.0E+06	40:1	211:1
12	Luciferase	2.0E+06	40:1	70:1
13	Luciferase	6.7E+05	13.4:1	35:1

^a^ PbTRAP-specific CD8^+^ cells: infected hepatocytes.

^b^ Sporozoite only wells were not included; number of infected hepatocytes was based on the median infectivity of 2.76%.

In order to limit the effect of non-specific killing, these splenocytes were then enriched for CD8^+^ T cells prior to addition into the *in vitro* assay (Fig. 4B). Given a previous report of such non-specific killing from naïve (unvaccinated mice) (22), in each experiment conducted CD8^+^ enriched splenocytes from vector control vaccinated mice were used as a control. The background inhibition from the vector control vaccinated mice did vary substantially between the independent experiments, from 0–70%, when compared to wells containing only sporozoites and no splenocytes (Fig. 4C and 4D). To account for the variation in background inhibition, the effect of addition of PbTRAP-specific CD8^+^ T cell enriched splenocytes was compared to the vector control in each experiment.

### Ability of *P*. *berghei* TRAP-specific CD8^+^ T cell enriched splenocytes to inhibit liver-stage parasites

**Fig 5:**
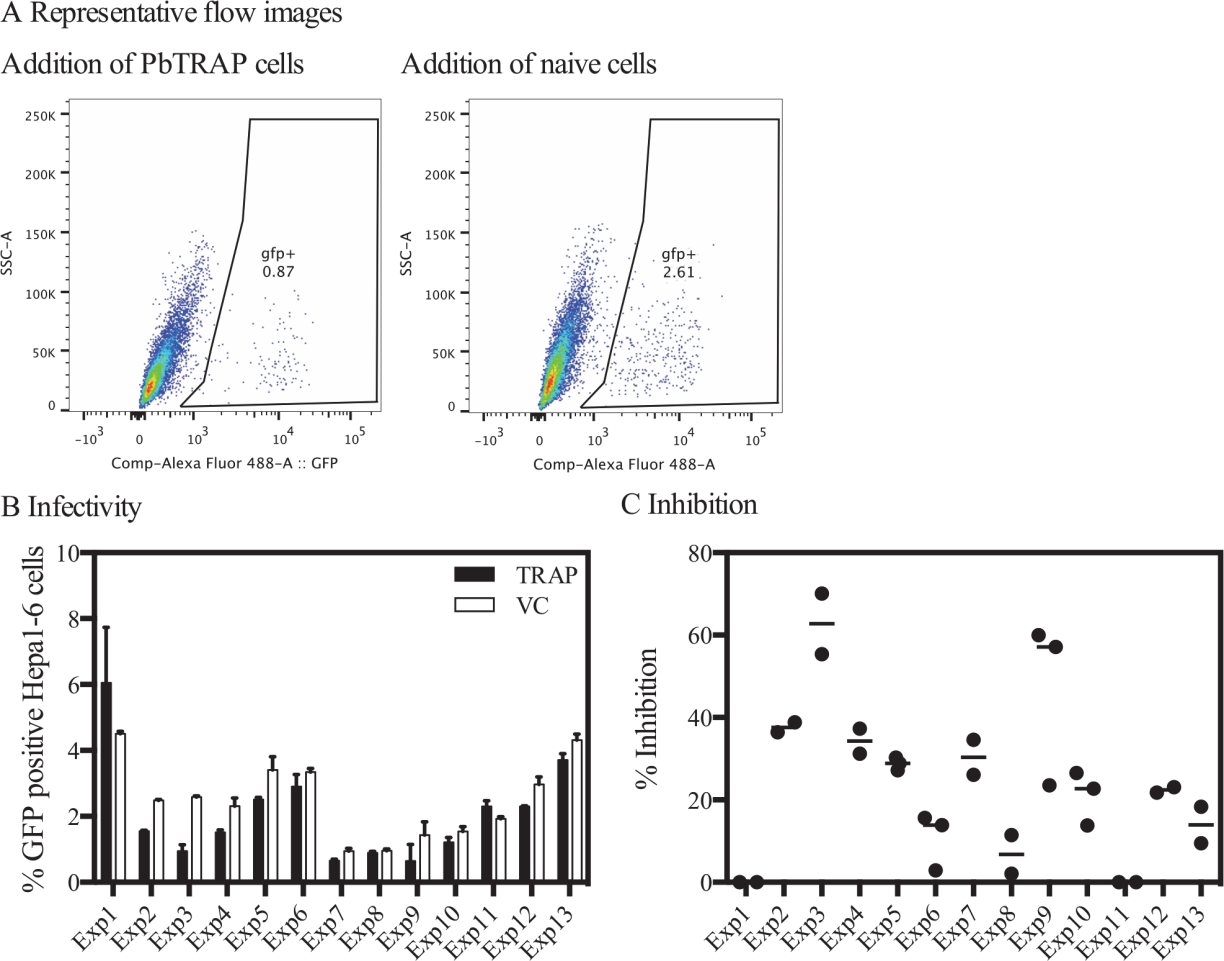
Inhibition of liver-stage parasites by *P*. *berghei* TRAP-specific CD8^+^ T cell enriched splenocytes. (A) Representative example of the flow cytometry plots, from ‘Experiment 3’ (see Table 1). (B) Results are expressed as the percentage infectivity, with the median shown for each experiment and error bars representing the interquartile range. A statistically significant difference was evident overall between wells containing splenocytes from PbTRAP compared to vector control vaccinated mice, p = 0.0479 (Wilcoxon matched-pairs signed rank test). (C) Results are expressed as the percentage inhibition compared to the wells containing cells from vector control vaccinated mice (equal number of CD8^+^ enriched splenocytes from ChAd63-MVA luciferase vaccinated mice), with median and individual data points shown for each experiment. If the percent inhibition was negative, it was considered to be zero.

Overall, the addition of PbTRAP-specific CD8^+^ T cell enriched splenocytes was shown to significantly reduce the number of liver-stage parasites compared with vector control wells, p = 0.0479 (Wilcoxon matched-pairs signed rank test) (Fig. 5A and B). This effect was seen in eleven out of thirteen experiments (Fig. 5B). However, the percentage inhibition achieved did vary between experiments (Fig. 5C), despite addition of the same number of splenocytes in the majority of experiments (Table 1). To account for changes in the frequency of PbTRAP-specific cells and purity of CD8^+^ enrichment between experiments, the E:T ratio was further defined as the number of antigen-specific CD8^+^ T cells (determined by ICS) to the number of infected hepatocytes (calculated from sporozoite only wells).

**Fig 6:**
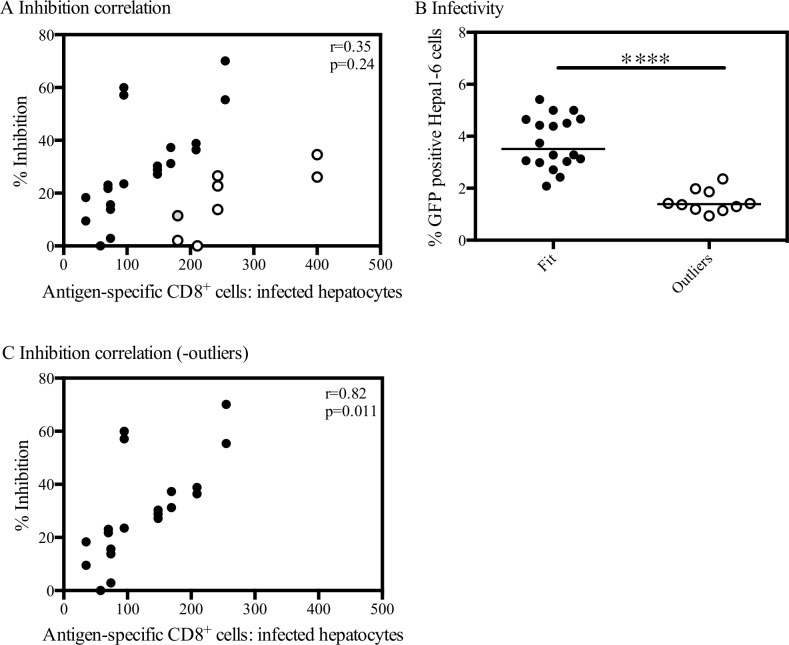
Correlation of the percentage inhibition with the effector to target ratio. (A) Results are expressed as the percent inhibition compared to mock-vaccinated control wells (equal number of CD8^+^ enriched splenocytes from ChAd63-MVA luciferase vaccinated mice). Across all thirteen experiments there was no significant correlation between E:T ratio and inhibition (Spearman r = 0.35, p = 0.24), although a trend can be observed. Full circles represent those experiments that fit this trend, whilst empty circles represent those that did not. Experiments were then divided into data that fitted the E:T pattern (full circles) and those that did not (empty circles) and a graph (B) of the infectivity measured in wells containing only Hepa1-6 cells and sporozoites are shown. Statistical difference was assessed using the Mann Whitney test, **** p<0.0001. (C) Correlation of the E:T ratio with the percentage inhibition (n = 9 experiments), excluding the outliers. Spearman r = 0.82, p = 0.011.

Addition of PbTRAP-specific CD8^+^ T cell enriched splenocytes was shown to inhibit liver-stage parasites in an E:T ratio dependent manner (Fig. 6A), however there was no statistically significant correlation observed (Spearman r = 0.35, p = 0.24). Of the thirteen independent experiments performed, four experiments did not fit the pattern observed (i.e. a greater number of effector cells were required to achieve similar levels of inhibition) and corresponded to significantly lower levels of infectivity (Mann Whitney test, p<0.0001) (Fig. 6B). When ‘outlier’ experiments were excluded, a statistically significant correlation between the percentage inhibition and the E:T ratio was observed (Spearman r = 0.82, p = 0.011) (Fig. 6C).

## Discussion

Currently, there is no standardized *in vitro* cellular assay to test the effectiveness of *P*. *falciparum* vaccine candidates in the pre-clinical phase of development or to assess immunological correlates of protection clinically. Here we have described the development of an improved murine *in vitro* assay and used it to demonstrate the ability of PbTRAP-specific CD8^+^ T cell enriched splenocytes to inhibit liver-stage parasites. The correlation between the number of PbTRAP-specific CD8^+^ T cells with the percentage inhibition, whilst not excluding the possibility that CD4^+^ T cells could have an effect, does suggests that CD8^+^ cells were the important effectors in our CD8^+^ enriched splenocyte suspension. This finding demonstrates the ability of PbTRAP to be processed and presented on the hepatocyte cell surface in association with MHC Class I molecules, in order to be recognized by CD8^+^ T cells. The only other antigen for which this has been demonstrated is CSP [21–23], and this could be how protection is mediated in the clinical TRAP vaccine [3].

To our knowledge, this is the first report of an *in vitro* cellular assay utilizing flow cytometry to measure infection based on GFP positivity. An added difficulty compared to antibody based assays is the need to distinguish between hepatocytes and effector T cells in order to reliably determine the percentage of GFP positive hepatocytes; we successfully utilized a membrane dye in order to stain and gate on hepatocytes only. A surprisingly high level of non-specific background inhibition was observed from vector control vaccinated mice; this is likely due to the presence of other cell types such as natural killer cells, and due to the large number of cells included in the assay. Calculating inhibition of PbTRAP vaccinated mice in comparison to infectivity of wells containing splenocytes from vector control vaccinated mice enabled us to normalize for this high level of background inhibition. The results suggested that a greater E:T ratio was required when a lower level of infectivity of hepatoma cells was achieved; this is likely an effect of the culture model and highlights that a standard level of infectivity will be required to compare results across experiments and between groups.

For the majority of our independent experiments we added 2x10^6^ total splenocytes, based on the total number of cells required to achieve the highest levels of cellular inhibition (60–80%) in *in vitro* assays previously reported [20, 22]. Our inhibition ranged from 0–60% and was associated not only with the effector-to-target ratio but also the level of infectivity of the hepatocytes. Comparatively, other groups have previously used a higher number of sporozoites to hepatocytes (varying from 1:1 to 5:1, compared to our 0.8:1) [21–24], and hence conceivably could have achieved higher levels of infection and hence inhibition, based on our current findings. Of those studies that achieved greater levels of inhibition with fewer total immune cells (Weiss *et al*. with 1x10^6^ immune cells [23], Renia *et al*. with 0.3x10^6^ immune cells [21], and Trimnel *et al*. with 0.05x10^6^ immune cells [24]), they were also using vaccination models that result in 100% protection (irradiated sporozoites or genetically attenuated parasites) or clonal cells, and hence it is not surprising that they outperformed the inhibition achieved with a sub-unit vaccine. Our current results are therefore physiologically relevant and highlight the value of this assay.

Apart from these aforementioned *in vitro* studies that have demonstrated the ability of T cells to inhibit liver-stage malaria parasites, two other experimental findings in murine models highlight the importance of CD8^+^ T cells: (i) adoptive transfer of CD8^+^ T cell clones specific for TRAP or CSP can induce protection against *P*. *berghei* and *P*. *yoelii* in naive mice [39–42] and (ii) depletion of CD8^+^ T cells completely abolishes the protection seen in radiation and genetically attenuated sporozoite models [24, 43–46]. Hence, there is a strong rationale for the development of a T cell-inducing malaria vaccine, and a need to be able to assess such vaccines based on *P*. *falciparum* antigens *in vitro*.

Whilst pre-clinical testing of vaccine candidates using murine models is widely accepted, there are a number of inherent limitations, most critically the limited MHC repertoire of inbred mice and the generation of single immunodominant epitopes. Hence, future work will aim to transfer the described *in vitro* assay from a murine-based system to an assay using *P*. *falciparum* sporozoites, primary human hepatocytes or cell lines and human T cells. Such an assay faces more difficulties: human leukocyte antigen (HLA) matching of human samples, the limited HLA type of available cell lines [47, 48] and the lower level of infectivity in human hepatocytes [48, 49], as well as the increased difficulty in obtaining *P*. *falciparum* sporozoites. However, these obstacles are not insurmountable.

Recently, cryopreserved primary human hepatocytes have become commercially available, enabling a new source of hepatocytes with a greater range of HLA types than cell lines. Furthermore, methods of culturing primary human hepatocytes have also improved [50], and such improvements are also expected to improve the subsequent infectivity. In addition, *P*. *falciparum* parasites expressing GFP are now available [51, 52], suggesting that direct transfer of our flow-cytometry based assay should be possible. The ability to cryopreserve *P*. *falciparum* sporozoites [53] will enable a reduction in the batch-batch variation in infectivity levels seen in the current study. The major difficulty in moving to a human assay will be inclusion of appropriate controls to account for the likely high non-specific background inhibition (based on our current findings). We suggest use of T cells stimulated with irrelevant antigens (such as influenza peptides), or the use of pre-vaccination T cells if evaluating the mechanisms of protection in clinical trials. Given many of the components of the human based assay have already been optimized and developed individually, and the remaining challenges can be addressed, the feasibility and utility of this assay is very promising.
